# Prognostic Value of Lymph-Node Micrometastases and Isolated Tumour Cells in Gastric Cancer

**DOI:** 10.7759/cureus.102007

**Published:** 2026-01-21

**Authors:** José Couto, Mariana Leite, João Mendes, Cláudia Lima, Inês Arnaud, Fábio Correia Viveiros, Ana Cristina Rodrigues

**Affiliations:** 1 General Surgery, Local Health Unit of Alto Minho, Viana do Castelo, PRT; 2 Surgery, Local Health Unit of Alto Minho, Viana do Castelo, PRT

**Keywords:** gastric cancer, immunohistochemistry staining, isolated tumour cells, micrometastases, perioperative chemotherapy

## Abstract

Background

Lymph node status is a crucial prognostic determinant in gastric cancer (GC). Conventional haematoxylin-eosin (H&E) assessment overlooks the micro volume of nodal disease. This study explored the prevalence and prognostic significance of lymph-node micrometastases (Mic) and isolated tumour cells (ITC) detected by cytokeratin AE1/AE3 immunohistochemistry (IHC) in patients undergoing gastrectomy with curative intent.

Methods

A retrospective, single-centre analysis of patients with gastric adenocarcinoma treated between 2014 and 2019 by curative intent gastrectomy with D2 lymphadenectomy and with IHC assessment of lymph nodes (LN). Nodal involvement was classified as macrometastases (>2 mm), Mic (>0.2-2 mm), or ITC (≤0.2 mm). Clinicopathological characteristics and survival outcomes were obtained from the institutional database. Data were analysed using χ²/Fisher's exact and Wilcoxon tests. Overall survival (OS) was analysed using Kaplan-Meier curves and log-rank tests.

Results

Eighty-six patients were included (mean age 67.2±11.2 years). A total of 1,974 lymph nodes were examined (mean 22.95±7.43 per patient): 256 (12.97%) contained macrometastases, 21 (1.06%) Mic and 31 (1.57%) ITC. Sixteen patients (18.6%) had Mic and/or ITC (ITC+/Mic+). Tumours larger than 20 mm with lymphovascular invasion were significantly associated with the presence of ITC+/Mic+. In node-negative (pN0) patients, IHC uncovered occult nodal disease in six cases (13.6%). In the overall cohort, five-year OS was 55.8%, and the presence of ITC+/Mic+ did not significantly affect OS across tumour, nodes, metastases (TNM) and pathological lymph node (pN) categories. In the subgroup treated with perioperative chemotherapy, the presence of ITC+/Mic+ was associated with a lower five-year OS (p=0.023). OS was comparable between pN0 patients with ITC+/Mic+ and those with node-positive (pN1) disease (p=0.624).

Conclusion

Micrometastases and ITC are detected in a relevant proportion of gastric cancer patients and are associated with adverse pathological features, but they did not independently influence OS in this clinical cohort. Their prognostic impact might be relevant in patients receiving perioperative chemotherapy. Routine IHC assessment is not sustained by these data and may be better suited for selected high-risk patients.

## Introduction

Lymph node metastasis is a key prognostic factor in gastric cancer (GC). The number of positive lymph nodes (LN) is essential for stage stratification and helps predict patient survival [1]. The current standard of care is radical gastrectomy, comprising gastric resection with adequate tumour-free margins and a D2 lymphadenectomy [2]. Available evidence demonstrates that radical gastrectomy with lymphadenectomy, resulting in node-negative (pN0) status on hematoxylin-eosin (H&E) staining, does not rule out the risk of recurrence [3,4]. Lymph node histopathological analysis depends on representative sections from resected nodes; consequently, lymph node micrometastases often escape routine detection. Recognition of this limited sensitivity has prompted the use of more sensitive assays to detect micrometastatic tumour cells.

With advances such as immunohistochemistry (IHC), the detection rate of micrometastases (Mic) or isolated tumour cells (ITC), not visible on routinely performed H&E staining, has increased [5-7]. Regional lymph node metastases could be subclassified based on the maximal tumour deposit dimension. Metastatic deposits measuring >0.2 to ≤2.0 mm are classified as micrometastases. Isolated tumour cells are defined as single cells or small clusters measuring <0.2 mm in greatest dimension [8]. Despite the growing research enabled by these methods, ITC and Mic have become an emerging area of investigation. Nevertheless, its clinical relevance remains debated, and there is no consensus on either optimal management or the prognostic impact [9]. To address this knowledge gap, we conducted a retrospective cohort study of patients undergoing curative-intent gastrectomy with D2 lymphadenectomy and cytokeratin AE1/AE3 immunohistochemistry assessment of negative lymph nodes. The primary objective was to evaluate the prognostic value of low-volume nodal disease, defined as isolated tumour cells and/or micrometastases, on overall survival. Secondary objectives were to quantify the prevalence of isolated tumour cells and micrometastases at both the patient and lymph-node levels and to identify clinicopathological features associated with them.

## Materials and methods

Study design and data collection

**Figure 1:**
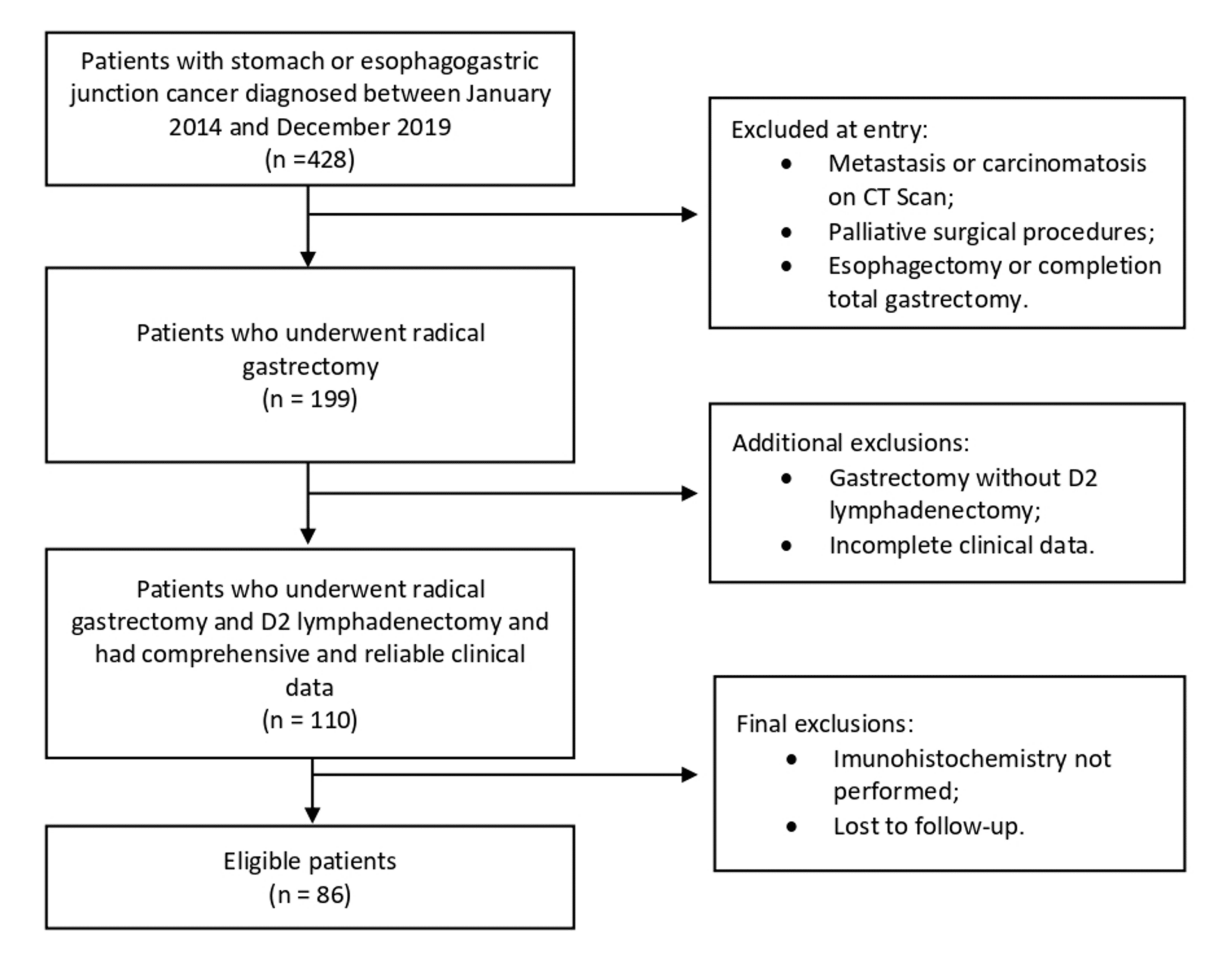
Flow chart showing the patient selection process

We analyzed gastric cancer patients treated with curative-intent gastrectomy between 2014 and 2019 at our institution. Patients were enrolled if they underwent radical gastrectomy with D2 lymphadenectomy, R0 margins, had no evidence of distant metastases, had complete clinicopathologic data available, and underwent immunohistochemical examination of lymph nodes (Figure 1). Of 428 patients diagnosed with gastric or oesophagogastric junction cancer (2014-2019), 199 underwent radical gastrectomy; 110 had D2 lymphadenectomy and comprehensive clinical data; and 86 met all inclusion criteria. Clinicopathological features, including tumour characteristics and overall survival (OS), were collected.

Lymph node evaluation

Immunohistochemistry Analysis

Lymph nodes retrieved were fixed in formalin and embedded in paraffin according to a standardized protocol. Routine histopathological examination with H&E was first conducted, and only cases classified as node-negative on conventional H&E were selected for supplementary immunohistochemical analysis, irrespective of the patient's overall pathological lymph node (pN) category. Lymph nodes already positive on H&E were not subjected to IHC.

For each lymph node, a single section was obtained at the mid-level (central portion) of the node, and two slides were prepared from this central section: one for routine H&E staining and, when applicable, a second slide for IHC.

IHC reassessment was performed using monoclonal antibodies against human cytokeratins (AE1/AE3). The AE1/AE3 antibody mixture targets human epithelial cytokeratins and shows no reactivity in lymphoid tissue. 

Tissue sections from paraffin blocks were deparaffinized with xylene and rehydrated with graded ethanol. Endogenous peroxidase activity was blocked with hydrogen peroxide for 30 minutes, followed by overnight incubation with the AE1/AE3 primary antibody at 4° C. The secondary antibody was then applied, followed by exposure to streptavidin-biotin peroxidase.

Positive expression was defined as cells displaying brownish-yellow granular staining within the cytoplasm.

Initial microscopic evaluation with H&E was performed by experienced gastrointestinal pathologists.

All immunohistochemically stained sections were evaluated by a single pathologist according to predefined histopathological criteria. Given the single-observer assessment, no formal interobserver agreement analysis was performed.

Definition of Lymph Node Positive Findings

Nodal disease was classified according to the maximal diameter of the metastatic deposit: macrometastasis (>2.0 mm), Mic (>0.2-2.0 mm), and ITC (single cells or clusters ≤0.2 mm). According to European Society for Medical Oncology (ESMO) and National Comprehensive Cancer Network (NCCN) guidelines, patients with isolated tumour ceIls or micrometastases in lymph nodes are staged as pN0. For comparative analyses, patients were categorized as ITC+ (presence of ITC), Mic+ (presence of micrometastasis), or ITC+/Mic+ (presence of ITC and/or micrometastasis) [10,11]. 

Statistical analysis

The descriptive analysis of qualitative variables was performed using absolute and relative frequencies; for quantitative variables, the mean and standard deviation were found. The comparison of means between groups was performed using the Wilcoxon non-parametric test. The association between categorical variables was evaluated by Fisher's exact test or the chi-square test, when appropriate. All tests were two-sided, and p-values less than 0.05 were considered statistically significant. Statistical analysis was performed using SPSS software, version 29.0 (IBM Inc., Armonk, US). This manuscript was prepared in accordance with the Strengthening the Reporting of Observational Studies in Epidemiology (STROBE) statement [12].

## Results

Patient and tumour characteristics

A total of 428 GC cases were reviewed, 86 met the inclusion criteria of curative-intent radical gastrectomy, R0 resection, and available immunohistochemical nodal assessment. The median follow-up duration was 50 months, and the five-year OS rate for the entire cohort was 55.8%. The mean age of the patients was 67.2±11.2 years, and 52.3% (n=45) were male. 

The vast majority of tumours (91.8%, n=79) were located in the middle or distal third of the stomach. According to Laurén's classification, the diffuse type was documented in 33.7% (n=29) of cases, followed by the intestinal type (60.5%, n=52) and the mixed type (5.8%, n=5).

Tumour was confined to the lamina propria of the mucosa in 2.3% (Tis, n=2), invaded the lamina propria, muscularis mucosae, or submucosa in 26.7% (T1, n=23), the muscularis propria in 19.8% (T2, n=17), the subserosa in 36.0% (T3, n=31), and the serosa in 15.1% (T4, n=13). 

Vascular, lymphatic, and perineural invasion were present in 24.4% (n=21), 24.4% (n=21), and 46.5% (n=40) of cases, respectively. Total gastrectomy was performed in 41.9% of patients and subtotal gastrectomy in 58.1% (n=50).

Nodal involvement profile

A total of 1974 lymph nodes were obtained and examined by routine H&E (mean 22.95±7.43 per specimen), whereas Mic and ITC were identified on H&E-negative nodes.

Overall, 256 (12.97%) held macrometastases, 21 (1.06%) Mic and 31 (1.57%) ITC. At the patient level, 44 patients (51.2%) were classified as pN0 on routine H&E, while 48.8% had macroscopic nodal involvement (pN1-pN3). Sixteen patients (18.6%) had either micrometastases or ITC identified by immunohistochemistry. 

Associations with clinicopathological features: risk factors for lymph node metastasis

The clinicopathologic characteristics of GC patients with and without Mic or ITC are summarised in Table 1.

**Table 1:** Prevalence according to the type of lymph node metastasis Prevalence of lymph-node tumour deposits according to metastatic deposit size. Results are presented as the number and proportion of lymph nodes containing isolated tumour cells (ITC, ≤0.2 mm), micrometastases (Mic, >0.2-2.0 mm), and macrometastases (>2.0 mm), out of all examined lymph nodes in the cohort.

Types of lymph node metastasis	n (%)
ITC	31 (1.57%)
Mic	21 (1.06%)
Macrometastasis	256 (12.97%)
Total (mean±SD)	1974 (22.95±7.43 )

No statistically significant differences were observed among the three cohorts: ITC+ (lymph nodes with isolated tumour cells only), Mic+ (lymph nodes with micrometastases only), and ITC+/Mic+ (lymph nodes with either isolated tumour cells or micrometastases). Baseline clinicopathological characteristics were comparable across cohorts, including age, sex, tumour location, histologic type, tumour differentiation grade, non-oncologic mortality, chemotherapy modality, and pathological tumour-node-metastases (pTNM) stage. 

Tumour size and lymphovascular invasion emerged as relevant risk factors: tumours >20 mm were significantly more likely to have ITC+/Mic+ nodes than tumours ≤20 mm (18.6% vs 2.3%, p=0.029). Lymphatic invasion and venous invasion were both associated with a higher frequency of ITC+/Mic+ (each p=0.035). ITC+/Mic+ was also associated with lower nodal categories (p=0.042).

**Table 2:** Association between the type of lymph node involvement and clinicopathologic features Clinicopathological characteristics stratified by occult nodal disease detected on AE1/AE3 immunohistochemistry. Patients are grouped as ITC+, Mic+, and ITC+/Mic+ (either ITC or Mic), and compared with those without occult nodal disease. P-values refer to between-group comparisons using χ²/Fisher's exact test for categorical variables and the Wilcoxon test for continuous variables, as appropriate. ITC - isolated tumour cells; Mic - micrometastases; pT - pathological tumour; pN - pathological lymph node; pTNM - pathological tumour-node-metastases; LN - lymph node; OS - overall survival

Characteristics	ITC+, n (%)	p-value	Mic+, n (%)	p-value	ITC+/Mic+, n (%)	p-value	Total
Age in years	72 (median)	0.543	68 (median)	0.62	68 (median)	0.906	67.16±11.23
Sex							
Male	6 (7%)	0.629	8 (9.3%)	0.284	10 (12.0%)	0.397	45 (52.3%)
Female	7 (8.1%)		4 (4.7%)		6 (7.2%)		41 (47.7%)
Chemotherapy modality							
Perioperative	4 (4.7%)	0.855	2 (2.3%)	0.548	5 (5.8%)	0.817	22 (25.6%)
Neoadjuvant	0 (0%)		0 (0%)		0 (0%)		2 (2.3%)
Adjuvant	3 (3.5%)		6 (7.1%)		7 (8.2%)		27 (31.4%)
No chemotherapy	6 (7.1%)		4 (4.7%)		6 (7.1%)		34 (40%)
Tumour localization							
Proximal	2 (2.3%)	0.468	2 (2.3%)	0.411	2 (2.3%)	0.71	5 (5.8%)
Middle	5 (5.8%)		5 (5.8%)		8 (9.3%)		39 (45.3%)
Distal	6 (7%)		5 (5.8%)		8 (9.3%)		40 (46.5%)
Greatest tumour dimension (mm)							
≤20	2 (2.3%)	0.207	1 (1.2%)	0.093	2 (2.3%)	0.029	28 (32.6%)
>20	11 (12.8%)		11 (12.8%)		16 (18.6%)		58 (67.4%)
pT							
pis	0 (0%)	0.448	0 (0%)	0.394	0 (0%)	0.199	2 (2.3%)
pT1	2 (2.3%)		3 (3.5%)		3 (3.5%)		23 (26.7%)
pT2	2 (2.3%)		2 (2.3%)		3 (3.5%)		17 (19.8%)
pT3	8 (9.3%)		7 (8.1%)		11 (12.8%)		31 (36%)
pT4	1 (1.2%)		0 (0%)		1 (1.2%)		13 (15.1%)
pN							
pN0	6 (7%)	0.432	3 (3.5%)	0.07	6 (7.0%)	0.042	44 (51.2%)
pN1	4 (4.7%)		7 (8.1%)		8 (9.3%)		17 (19.8%)
pN2	0 (0%)		1 (1.2%)		1 (1.2%)		9 (10.5%)
pN3	3 (3.5%)		1 (1.2%)		3 (3.5%)		16 (18.6%)
pTMN							
pTMN, stage I	3 (3.5%)	0.397	3 (3.5%)	0.278	4 (4.7%)	0.129	31 (36.0%)
pTMN, stage II	6 (7%)		8 (9.3%)		10 (11.6%)		29 (33.7%)
pTMN, stage III	3 (3.5%)		1 (1.2%)		3 (3.5%)		20 (23.3%)
pTMN, stage IV	0 (0%)		0 (0%)		0 (0%)		1 (1.2%)
Type of invasion							
Lymphatic	6 (7%)	0.75	6 (7%)	0.063	8 (9.3%)	0.035	21 (24.4%)
Venous	5 (5.8%)	0.291	7 (8.1%)	0.007	8 (9.3%)	0.035	21 (24.4%)
Perineural	8 (9.3%)	0.238	8 (9.3%)	0.131	11 (12.8%)	0.163	40 (46.5%)
Laurén classification							
Intestinal	6 (7.0%)	0.161	6 (7.0%)	0.192	8 (9.3%)	0.16	52 (60.5%)
Diffuse	5 (5.8%)		4 (4.7%)		8 (9.3%)		29 (33.7%)
Mixed	2 (2.3%)		2 (2.3%)		2 (2.3%)		5 (5.8%)
Differentiation							
Well differentiated (G1)	1 (1.2%)	0.320	0 (0%)	0.208	1 (1.2%)	0.164	15 (17.4%)
Moderately differentiated (G2)	4 (4.7%)		5 (5.8%)		6 (7.0%)		34 (39.5%)
Poorly differentiated (G3)	8 (9.3%)		7 (8.1%)		11 (12.8%)		37 (43.0%)
Operative procedure							
Total gastrectomy							36 (41.9%)
Distal gastrectomy							50 (58.1%)
Anastomosis							
BII							44 (51.2%)
Y-Roux							42 (48%)
Mean of LN retrieved							22.95 (±7.43)
Five-year OS	2 (2.8%)	1	3 (4.2%)	0.847	4 (5.6%)	1	22 (30.6%)
Non-oncologic mortality							14 (16.3%)
Patients demise due to postoperative complications							5 (5.8%)
Death related to chemotherapy							2 (2.3%)

Overall cohort

**Figure 2:**
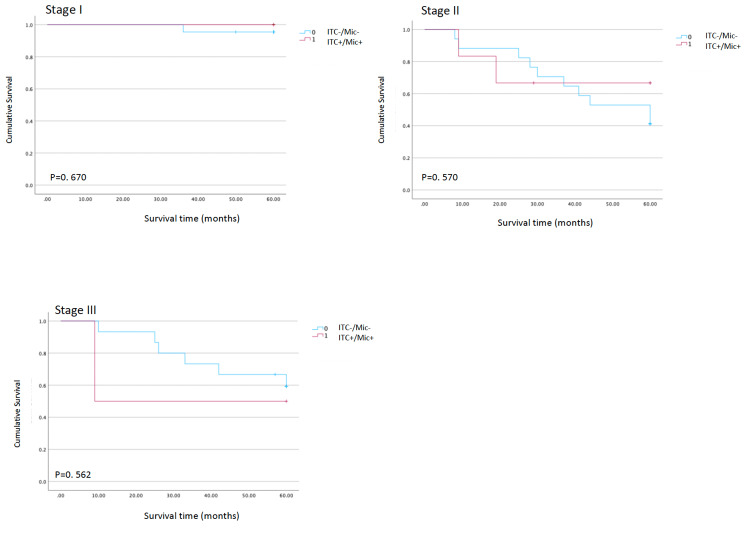
Test of equality of survival distributions for the presence of ITC or Mic in different stages Kaplan-Meier overall survival curves comparing patients with occult nodal disease (ITC+/Mic+) versus those without occult nodal disease (ITC-/Mic-), stratified by pathological tumour-node-metastases (TNM) stage (I–III). P-values shown in each panel correspond to log-rank tests comparing survival distributions between groups within each stage. ITC - isolated tumour cells; Mic - micrometastases

**Figure 3:**
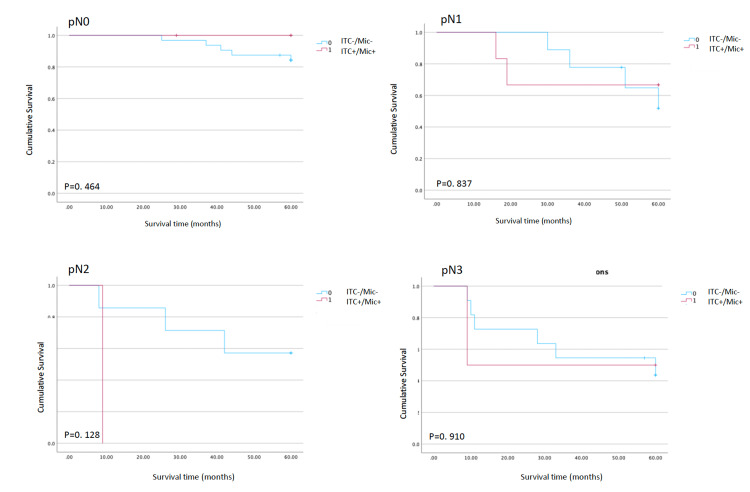
Test of equality of survival distributions for the presence of ITC or Mic in different pN stages Kaplan-Meier overall survival curves comparing patients with occult nodal disease (ITC+/Mic+) versus those without occult nodal disease (ITC-/Mic-), stratified by pathological nodal category (pN0-pN3). Group differences were assessed using log-rank tests, with p-values reported for each pN stratum. ITC - isolated tumour cells; Mic - micrometastases; pN0 - node-negative; pN0-pN3 - node-positive

Kaplan-Meier analysis showed no significant difference in OS between patients with (ITC+/Mic+) and without micrometastases (ITC-/Mic-). The overall five-year recurrence rate was 30.6%. When patients were stratified by pathological TNM stage, the presence of ITC+/Mic+ did not significantly affect OS in any subgroup (Figure 2). Similarly, when survival was analysed according to pathological nodal status (pN0-pN3), ITC/Mic status had no significant impact on OS (pN0: p=0.464; pN1: p=0.837; pN2: p=0.128; pN3: p=0.910; Figure 3).

pN0 and perioperative subgroups

Among the 44 patients classified as pN0 on conventional histology, cytokeratin immunostaining revealed occult nodal disease in six patients (13.6% of pN0; 7% of the entire cohort). In this group, 20 of 946 lymph nodes initially reported as negative (2.11%) were found to contain tumour deposits (six nodes with micrometastases and 14 with ITC).

**Figure 4:**
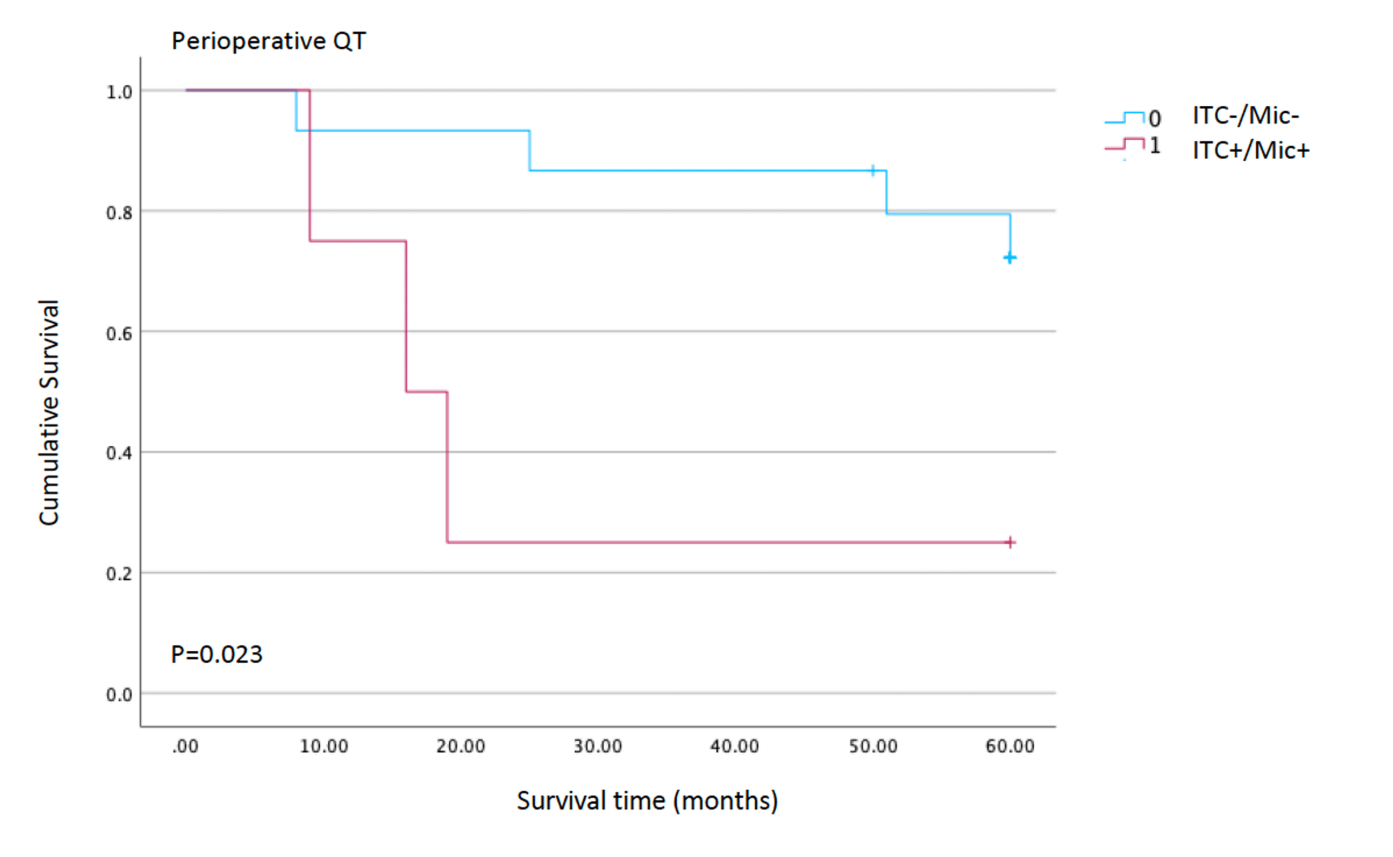
Test of equality of survival distributions for the presence of ITC or Mic with perioperative chemotherapy Kaplan-Meier curves in the subgroup treated with perioperative chemotherapy, comparing outcomes between patients with occult nodal disease (ITC+/Mic+) and those without occult nodal disease (ITC-/Mic-). The log-rank test indicates a significant difference between curves (p=0.023), suggesting an association between occult nodal disease and higher relapse risk in this subgroup. ITC - isolated tumour cells; Mic - micrometastases

A subset of patients who received perioperative chemotherapy observed a significant association between occult nodal disease and OS. In these patients, the presence of micrometastatic nodal involvement detected by immunohistochemistry was associated with a higher recurrence rate (p=0.023; Figure 4), suggesting a potential prognostic role for ITC/Mic status.

Comparison of pN0 with ITC+/Mic+ vs pN1

The Kaplan-Meier analysis comparing OS between patients with pN0 disease with ITC+/Mic+ and those with pN1 nodal involvement shows largely overlapping survival curves throughout the follow-up period. Both groups maintain estimated survival above roughly 70% at five years, and no clear separation of the curves is observed.

**Figure 5:**
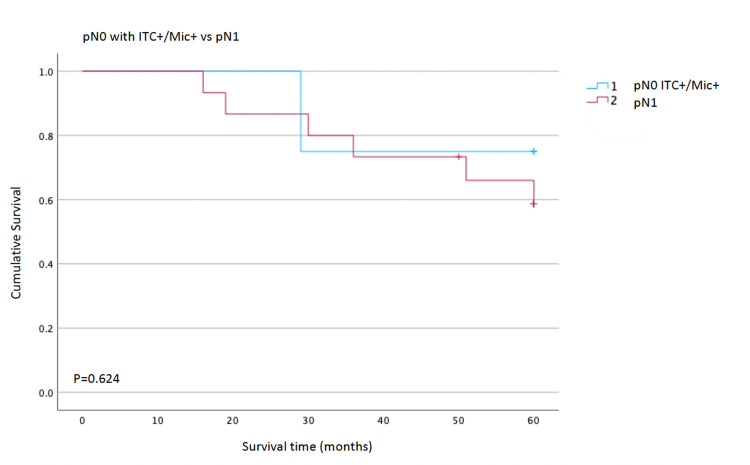
Test of equality of survival distributions for the presence of ITC or Mic in pN0 and pN1 Kaplan-Meier overall survival curves comparing patients classified as pN0 on conventional histology but harbouring occult nodal disease on immunohistochemistry (pN0 with ITC+/Mic+) versus patients with overt pN1 disease. Survival distributions were compared using a log-rank test (p=0.624), showing no statistically significant difference between groups. ITC - isolated tumour cells; Mic - micrometastases; pN0 - node-negative; pN1 - node-positive

There was no statistically significant difference in survival between the two groups (p=0.624, Figure 5), indicating that patients with micrometastatic nodal disease classified as pN0 do not experience significantly different overall survival compared with patients classified as pN1.

## Discussion

In this single-centre cohort, we observed that micrometastases and ITC affected a non-negligible proportion of patients. Sixteen patients (18.6%) had either micrometastases or ITC, indicating that a relevant subset of patients carried occult nodal tumour cells detectable exclusively by IHC.

In patients staged as pN0 by routine histology, cytokeratin immunostaining revealed previously unidentified tumour cells in six cases (13.64%), corresponding to 7% of the overall cohort. These findings are somewhat lower than the 20-30% prevalence of micrometastases reported in several series that employed serial sectioning, but they are consistent with the wide range observed in the literature and likely reflect methodological differences, including the number of sections per node [13,14].

Consistent with previous reports, we identified specific histopathological features associated with micrometastatic disease. Tumour size greater than 20 mm and the presence of lymphovascular invasion were significantly associated with a higher prevalence of micrometastatic lymph-node involvement. The proportion of patients with ITC+/Mic + was 18.6% among tumours >20 mm compared with 2.3% among smaller tumours. These findings support the concept that micrometastatic spread is not a random event but rather a marker of a more biologically aggressive phenotype marked by enhanced lymphovascular dissemination.

Despite this, our survival analysis did not demonstrate a clear adverse impact of these cells on overall prognosis in the study population as a whole. Kaplan-Meier curves showed no significant difference in OS between patients with and without lymph node metastasis, either when stratified by pathological TNM stage or by nodal category (pN0-pN3). These observations place our results closer to those series in which micrometastases were not identified as a prognostic factor and contrast with meta-analytic data suggesting a consistently negative impact of micrometastatic disease on long-term outcomes [15].

A potentially clinically relevant result was observed from the subgroup of patients treated with perioperative chemotherapy. In this subset, the presence of Mic or ITC was associated with a significantly lower OS (p=0.023), suggesting that occult nodal disease might have prognostic value in these patients. One possible explanation is that micrometastatic involvement identifies a subgroup with higher systemic dissemination potential, in whom perioperative chemotherapy may be insufficient to eradicate residual nodal disease. Alternatively, this association may partly reflect selection bias and the small number of events. We also compared survival between patients with pN0 disease with ITC+/Mic + and those with overt pN1 nodal involvement. Interestingly, Kaplan-Meier curves showed substantial overlap, and the log-rank test revealed no significant difference in OS between these groups (p=0.624). Although our study was underpowered to draw definitive conclusions, this finding suggests that micrometastatic nodal disease may, in prognostic terms, behave more similarly to limited macrometastatic involvement (pN1) than to truly node-negative disease. This observation is consistent with the stage-migration hypothesis proposed in other series, in which the inclusion of micrometastases in the count of positive nodes tends to align survival more closely with higher N categories [14-15]. From a surgical perspective, our data confirm the feasibility of achieving adequate nodal retrieval in routine practice, with a mean of approximately 23 lymph nodes examined per specimen, in accordance with D2 lymphadenectomy standards. A small proportion of lymph nodes exhibit occult tumour deposits detectable only by IHC, underscoring the intrinsic limitations of single-section H&E evaluation. Nevertheless, given that micrometastases and ITC did not significantly affect OS in the overall cohort, routine immunohistochemical assessment of all patients with gastric cancer does not appear justified based on our results.

The present study has several limitations that must be acknowledged. It is a retrospective, single-centre study with a relatively small sample size, which limits statistical power, particularly in subgroup analyses. The number of patients with ITC+/Mic+ and the number of recurrences were modest, increasing the risk of type II error and potentially masking subtle prognostic effects. Furthermore, our methodology did not include exhaustive serial sectioning of all lymph nodes, which may have led to underestimation of other small deposits. Despite these limitations, our findings contribute to the existing literature by providing contemporary data from a Western cohort treated with radical gastrectomy with D2 surgery and perioperative therapy.

Considering all available evidence, our study results suggest that, while micrometastases and ITC are biologically meaningful and associated with adverse histopathological features, their prognostic impact on long-term survival may be limited. Their role may be more subtle, potentially refining risk stratification in specific subgroups, such as those receiving perioperative chemotherapy, rather than serving as a universal indicator of poor prognosis.

## Conclusions

Micrometastases and isolated tumour cells were detected in nearly one-fifth of patients, most often in association with larger tumours and lymphovascular invasion. However, their presence did not affect overall survival in the cohort, and outcomes in pN0 patients with micrometastases were similar to those of pN1 patients. A prognostic effect was observed exclusively in patients receiving perioperative chemotherapy, in whom micrometastases or ITC were linked to a lower OS. This technique appears to reveal features of a more aggressive tumour phenotype but, by itself, does not justify routine use. Prospective, standardised studies are needed to clarify their role in staging and treatment decision-making.
